# Synthesis of BaSiH_6_ Hydridosilicate at High Pressures—A Bridge to BaSiH_8_ Polyhydride

**DOI:** 10.1021/acsomega.4c10502

**Published:** 2025-04-07

**Authors:** Doreen C. Beyer, Kristina Spektor, Olga Yu Vekilova, Jekabs Grins, Paulo H. Barros Brant Carvalho, Logan J. Leinbach, Michael Sannemo-Targama, Shrikant Bhat, Volodymyr Baran, Martin Etter, Asami Sano-Furukawa, Takanori Hattori, Holger Kohlmann, Sergei I. Simak, Ulrich Häussermann

**Affiliations:** † Institute for Inorganic Chemistry and Crystallography, Leipzig University, Johannisallee 29, D-04103 Leipzig, Germany; ‡ Deutsches Elektronen-Synchrotron DESY, Notkestraße 85, D-22607 Hamburg, Germany; § Department of Materials and Environmental Chemistry, Stockholm University, SE-10691 Stockholm, Sweden; ∥ Department of Chemistry−Ångström, Uppsala University, SE-75121 Uppsala, Sweden; ⊥ Eyring Materials Center, Arizona State University, Tempe, Arizona 85287, United States; # J-PARC Center, Japan Atomic Energy Agency, 2-4 Shirakata, Tokai-mura, Naka-gun, Ibaraki 319-1195, Japan; ∇ Theoretical Physics Division, Department of Physics, Chemistry and Biology (IFM) Linköping University, SE-581 83 Linköping, Sweden; ○ Department of Physics and Astronomy, Uppsala University, SE-75120 Uppsala, Sweden

## Abstract

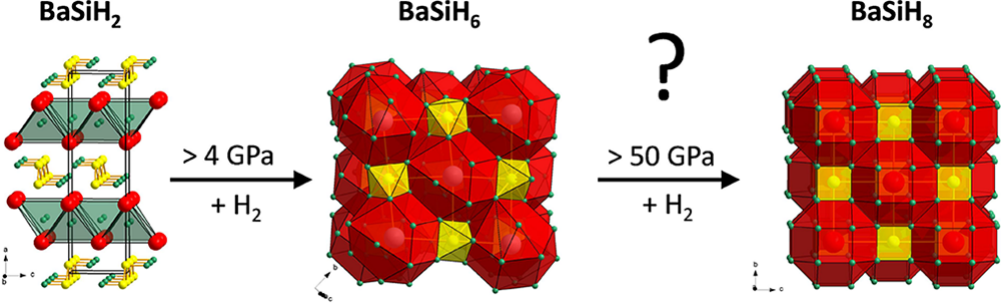

Hydridosilicates featuring SiH_6_ octahedral moieties represent a rather new class of compounds with potential properties relating to hydrogen storage and hydride ion conductivity. Here, we report on the new representative BaSiH_6_ which was obtained from reacting the Zintl phase hydride BaSiH_∼1.8_ with H_2_ fluid at pressures above 4 GPa and subsequent decompression to ambient pressure. Its monoclinic crystal structure (*C*2/*c*, *a* = 8.5976(3) Å, *b* = 4.8548(2) Å, *c* = 8.7330(4) Å, β = 107.92(1)°, *Z* = 4) was characterized by a combination of synchrotron radiation powder X-ray diffraction, neutron powder diffraction, and DFT calculations. It consists of complex SiH_6_^2–^ ions (*d*_Si–H_ ≈ 1.61 Å), which are octahedrally coordinated by Ba^2+^ counterions. The arrangement of Ba and Si atoms deviates only slightly from an ideal fcc NaCl structure with *a* ≈ 7 Å. IR and Raman spectroscopy showed SiH_6_^2–^ bending and stretching modes in the ranges 800–1200 and 1400–1800 cm^–1^, respectively, in agreement with a hypervalent Si–H bonding situation. BaSiH_6_ is thermally stable up to 95 °C above which decomposition into BaH_2_ and Si takes place. DFT calculations indicated a direct band gap of 2.5 eV and confirmed that at ambient pressure BaSiH_6_ is a thermodynamically stable compound in the ternary Ba–Si–H system. The discovery of BaSiH_6_ consolidates the compound class of hydridosilicates, accessible from hydrogenations of silicides at gigapascal pressures (<10 GPa). The structural properties of BaSiH_6_ suggest that it presents an intermediate (or precursor) for further hydrogenation at considerably higher pressures to the predicted superconducting polyhydride BaSiH_8_ [Lucrezi, R.; et al. *npj Comput. Mater.***2022**, *8*, 119] whose structure is also based on a NaCl arrangement of Ba and Si atoms but with Si in a cubic environment of H.

## Introduction

Recently, it has been shown that silicon is capable of forming hydrogen-rich hydridosilicates featuring octahedral SiH_6_^2–^ complex ions. Synthesis efforts until today yielded K_2_SiH_6_, Rb_2_SiH_6_, and Na_3_SiH_7_.^1−3^ More potential representatives, like Na_2_SiH_6_, NaSiH_5_, Li_2_SiH_6_, have been proposed from computational structure prediction.^3−6^ Hydridosilicates are salt-like compounds and their structural chemistry resembles that of well-established fluorosilicates/germanates. However, in contrast with fluorosilicates, hydridosilicates are not insulators but semiconductors. The occurrence of hypervalent SiH_6_^2–^ moieties is peculiar since the polarity of the Si^δ+^–H^δ−^ bond is rather small and hypervalence of p-elements is usually associated with electronegative ligands, such as F. It has been suggested that hydridosilicates may have interesting hydride ion conducting and/or hydrogen storage properties,^5^ yet the physical, chemical, and materials properties of these compounds remain poorly investigated because of their scarcity.

The synthesis of hydridosilicates typically requires high pressure conditions in the multi GPa range, 4–10 GPa.^1−3^ This pressure range is accessible with large-volume press (LVP) high-pressure methodology which provides sizable sample volumes and well-controlled *p*, *T* environments for high-pressure hydrogenation reactions.^7−9^ In LVP hydrogenations, H_2_ has to be delivered by an internal solid source which is integrated in the sample. Ammonia borane, BH_3_NH_3_, has been recognized as an ideal H-source as it possesses a high H content and decomposes neatly to inert BN and H_2_ at comparatively low temperatures (200–400 °C, depending on *p*).^10^ Various types of precursor scenarios may be considered for high pressure hydrogenations potentially leading to hydridosilicates, such as *M*H_*n*_ + Si, *M*_*m*_Si_*n*_, *M*_*m*_Si_*n*_H_*x*_ (*M* = active metal), of which the latter, *M*_*m*_Si_*n*_ (silicide Zintl phases) and *M*_*m*_Si_*n*_H_*x*_ (Zintl phase hydrides), have shown largely superior kinetics^2^ thus enabling lower synthesis temperatures and potentially access to metastable products.

We have started to explore more systematically the accessibility and compositional variety of hydridosilicates. Here we report on the synthesis of BaSiH_6_ from hydrogenation of the Zintl phase hydride BaSiH_2–*x*_ at ∼7 GPa which resulted in sizable bulk samples with several tens of mg quantities. We find that the structure of BaSiH_6_ closely relates to that of the even more hydrogen-rich metallic and high *T*_C_ superconducting polyhydride BaSiH_8_ which has been predicted to be thermodynamically stable at ultrahigh pressures, above 100 GPa.^11^

## Methods

### Synthesis of BaSiH_2–*x*_ (*x* ≈ 0.15) Precursor

The Zintl phase hydride BaSiH_2–*x*_ was prepared by sintering BaSi in a hydrogen atmosphere (H_2_, Air Liquide, 99.9%) at 90 bar pressure and 180 °C for 24 h using an autoclave made from H-resistant Nicrofer 5219Nb alloy.^12^ BaSi in turn was synthesized by arc-melting mixtures of the elements (Si, ChemPur, 99.9999%; Ba rod, 99.3%) with a slight (about 5%) Ba excess. The synthesis of the deuteride BaSiD_2–*x*_ is described in the Supporting Information section.

### Synthesis of BaSiH_6_

All steps of sample preparation for high pressure (LVP) hydrogenation experiments were performed in an Ar-filled glovebox. NaCl is considered an ideal capsule material since it provides tight seals and resists hydrogen diffusion.^7−9^ Powdered precursor BaSiH_2–*x*_ and hydrogen source BH_3_NH_3_ (Sigma Aldrich, 97%) were pressed into pellets with an outer diameter (OD) of 4 mm and ∼3 and ∼1.3 mm height, respectively. A precursor pellet was placed between two BH_3_NH_3_ pellets and sealed in a NaCl capsule (OD 6.1 mm, ∼7.8 mm height). The molar ratio BaSiH_2–*x*_:BH_3_NH_3_ corresponded roughly to 1:1 (BaSiH_2–*x*_:H_2_ = 1:3). Two synthesis runs were conducted at Arizona State University (ASU) using the COMPRES 18/12 assemblies.^13^ Samples were compressed in a 1000 tonnes press to ∼7 GPa and subsequently heated to 550 °C over two hours, held at this temperature for eight hours, cooled back to room temperature over 1 h, and subsequently decompressed. The temperature was measured with a type C thermocouple. Samples were recovered in a glovebox. In situ diffraction experiments were performed at various pressures between 4 and 19 GPa at the synchrotron facility PETRA III, DESY and at 5 GPa at the neutron facility MLF, J-PARC, employing the LVP beamlines P61B^14^ and PLANET,^15^ respectively. Results from these experiments will be reported separately. The J-PARC experiment, however, is described in more detail in the SI section.

### Powder X-ray Diffraction (PXRD)

PXRD patterns of recovered BaSiH_6_ were collected at the beamline P02.1, PETRA III, DESY^16^ using monochromatic synchrotron radiation (*E* ≈ 60 keV, λ = 0.20734–0.20738 Å). Samples produced at P61B were sealed (as sintered pieces without grinding) inside 1.0 mm diameter glass capillaries. For multitemperature measurements, the sample produced at ASU was finely ground and sealed in 0.3 mm diameter fused silica capillaries. Capillaries were heated with a mini hot air blower which is part of the sample environment at P02.1. For indexing of diffraction patterns, the Crysfire package^17^ was used. Le Bail analysis and Rietveld refinement of the PXRD data were performed in Jana2006.^18^ Further details are provided as SI.

### Spectroscopy

FTIR spectra were recorded with a “Golden Gate” micro-ATR accessory using a Varian IR-670 spectrometer with a thermostatted DTGS detector. A powdered sample of BaSiH_6_ was transferred from the glovebox in an airtight vial. At the spectrometer, the vial was opened, and the powder was quickly clamped between the ATR diamond and sapphire elements. During the transfer process, moisture-sensitive BaSiH_6_ may have decomposed slightly. For Raman spectroscopy, powdered samples were sealed in 0.3 mm Lindemann capillaries. Spectra were measured using a LabRAM HR 800 spectrometer. The instrument is equipped with an 800 mm focal length spectrograph and a Peltier-cooled (−70 °C), back-thinned CCD detector. Samples were excited using a double-frequency Nd:YAG laser (532 nm). A 10% filter was applied corresponding to a low power density of 5.5 × 10^–6^ mW·μm^–2^). Raman spectra were collected with an exposure time of 150 s (100 accumulations) and using a 600 grooves/mm grating.

### Theoretical Calculations

Crystal structure prediction (CSP) utilizing the USPEX code^19−21^ coupled with the Vienna Ab Initio Simulation Package (VASP)^22,23^ was employed for assisting the identification of the monoclinic BaSiH_6_ structure. For the USPEX calculation, we used the numerical constraints of a fixed composition (1:1:6) and the maximum number of atoms in a candidate cell of 32 (*Z* = 4). The lowest enthalpy structure identified had *C*2/*c* space group symmetry and *Z* = 4. VASP calculations were based on a first-principles projector-augmented wave method^24^ within the density functional theory (DFT).^25^ The generalized gradient approximation for exchange and correlation potential and energy was used in its Perdew–Burke–Ernzerhof (PBE) flavor.^26,27^ Total energy/electronic structure calculations were performed for BaH_2_, BaSiH_2_, Si, and BaSiH_6_ on fully relaxed structures. Hydrogen was calculated as a molecule H_2_. The Monkhorst–Pack^28^*k*-point density for integrations over the Brillouin zone was set with 0.2. The energy cutoff for plane waves was 800 eV. Phonon dispersions were calculated with PHONOPY.^29^ The required dynamical matrix was obtained via highly accurate calculations with density functional perturbation theory as implemented in VASP, with the energies converged down to 10^–8^ eV.

## Results and Discussion

**Figure 1:**
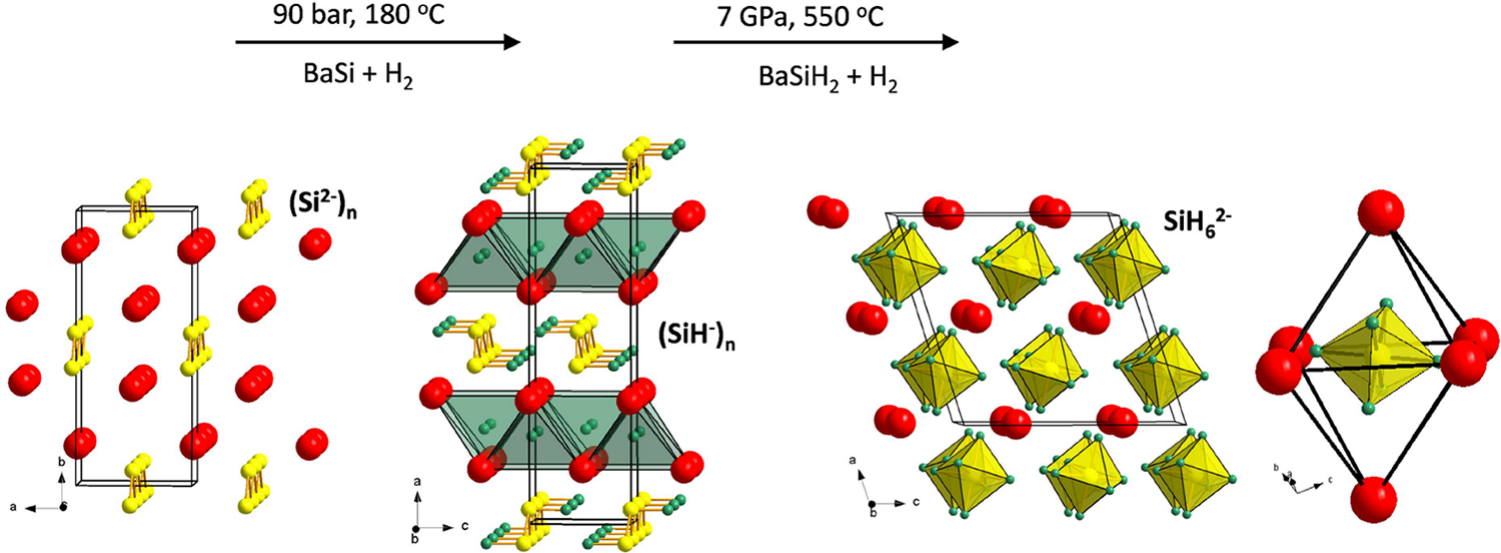
From left to right: low pressure hydrogenation transforms the Zintl phase BaSi (CrB structure type, *Cmcm*, *Z* = 4) into the hydride BaSiH_2–*x*_ (*x* ≈ 0.15; *Pnma*, *Z* = 4),^12^ which in turn yields BaSiH_6_ (*C*2/*c*, *Z* = 4) upon hydrogenation at gigapascal pressures. Ba, Si, and H atoms are depicted as red, yellow, and green circles, respectively. Far right: octahedral coordination of a SiH_6_^2–^ ion by six Ba^2+^ counterions in *C*2/*c* BaSiH_6_ (based on DFT relaxed H atom positions, see text).

The Zintl phase BaSi (CrB structure type) features polyanionic zigzag chains of Si atoms (^1^_∞_[Si^2–^]).^30^ Upon hydrogenation at low-pressure autoclave conditions (90 bar (9 MPa)), the Zintl phase hydride BaSiH_2–*x*_ is obtained for which the basic structural features of BaSi are retained.^12^ In the structure of BaSiH_2–*x*_, H is located as a hydride (H^–^) anion in tetrahedral interstices formed by the Ba atoms. In addition, the zigzag chain Si atoms are decorated with covalently bonded H atoms (thus changing ^1^_∞_[Si^2–^] into a ^1^_∞_[SiH^–^] polyanion), Figure 1. However, the site of the H coordinating Si has a slight deficiency of about 15%. Si atoms in ^1^_∞_[SiH^–^] obey the octet rule, and stoichiometric BaSiH_2_ would correspond to an electronically balanced Zintl phase, i.e., Ba^2+^(SiH)^−^(H^–^).^12^

**Figure 2:**
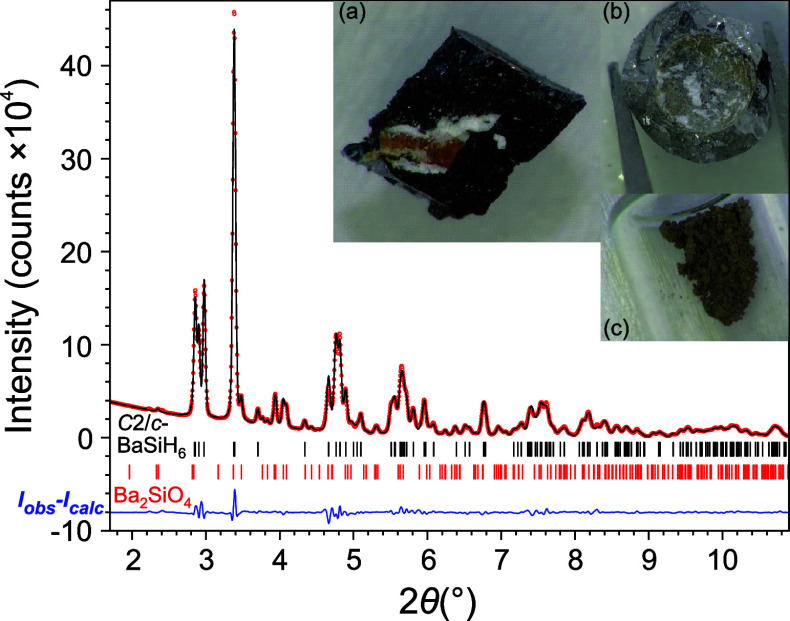
Rietveld fit of the BaSiH_6_ structure to a synchrotron PXRD pattern (λ = 0.20734 Å). Vertical lines are reflection markers for BaSiH_6_ and Ba_2_SiO_4_ impurity. Phase fractions: ∼84(1) wt% (92 mol%) BaSiH_6_ and ∼16(1) wt% (8 mol%) Ba_2_SiO_4_. Inset (a) shows a photograph of an orange-colored BaSiH_6_ sample from an in situ experiment (∼8.5 GPa, 570 °C) before recovery, embedded between BN residual from decomposed BH_3_NH_3_ inside the NaCl capsule (which attained a black color due to X-ray irradiation). Note that the sample is largely rearranged from its original position during the reaction due to the escape of excess H_2_ upon decompression. Inset (b) shows a partially recovered pellet (4 mm diameter) of BaSiH_6_ from a scaled-up synthesis experiment (the NaCl capsule is partly broken off, and the surface of the pellet is not yet cleaned completely from BN residual), and inset (c) shows a part of this pellet ground into a fine powder inside a vial with a 6 mm diameter. The light brownish/gray color in (b) and (c) is attributed to a small grain size and/or the presence of Si impurity.

The formation of the hydridosilicate BaSiH_6_ was initially observed in in situ diffraction experiments when attempting the hydrogenation of BaSiH_2–*x*_ in the pressure interval 4–13 GPa, and from these experiments, it could also be shown that BaSiH_6_ is retained at ambient pressure (Figure 2). Subsequently, dedicated experiments were performed at 7 GPa and 550 °C to scale up its preparation, which for the first time yielded sizable quantities (several tens of mg) of a hydridosilicate. However, although the conversion of BaSiH_2–*x*_ proceeded quantitatively, all samples (originating from the same batch of BaSiH_2–*x*_) contained Ba_2_SiO_4_ (orthosilicate) as an impurity (3–8 mol%) which presumably originates from an oxide/peroxide/hydroxide contamination of the BaSiH_2–*x*_ precursor (cf. discussion in the SI section). BaSiH_6_ displays a reddish-orange color as well sintered specimen, but when it is small-grained or as powder, it appears light brownish/gray (insets in Figure 2).

The SR-PXRD pattern of BaSiH_6_ could be indexed to the monoclinic *C*-centered cell with *Z* = 4 that was as suggested from computational CSP (see SI for details). The refinement of the CSP model proceeded highly satisfactorily. Unambiguous confirmation of the H positions came then from an (in situ) neutron diffraction experiment, probing the formation of BaSiD_6_ at around 5 GPa (see SI for details), which provided a data set at 2.5 GPa and room temperature. To arrive at the H position parameters for BaSiH_6_ at ambient conditions, it was deemed most consistent to extract them from a DFT relaxation where the lattice parameters and Ba and Si atomic position parameters were constrained to the experimentally refined ones, referring to room temperature and ambient pressure. The resulting structure of BaSiH_6_ after the refinement is reported in Tables 1 and 2 (see also Figure 2 and SI section). It resembles closely the unique KPF_6_-III structure, which apparently was determined over 30 years ago^31^ but has come to attention only recently.^32^

**Table 1:** Results of the Rietveld Refinement of the BaSiH_6_ Structure at Ambient Conditions

crystal system	monoclinic
space group	*C*2/*c* (no. 15)
*Z*	4
lattice parameters^a^	*a* = 8.5976(3) Å
	*b* = 4.8548(2) Å
	*c* = 8.7330(4) Å
	β = 107.918(4)°
*V* (Å^3^)	346.84(2)
formula weight (g/mol)	171.46
*d*_calc_ (g/cm^3^)	3.284
*R*_obs_ (%)	1.39
*R*_all_ (%)	1.39

a Pseudocubic triclinic cell: *a* = 7.040 Å, *b* = 7.008 Å, *c* = 7.040 Å, α = 89.32°, β = 87.20°, γ = 89.32°.

**Table 2:** Fractional Coordinates and Atomic Displacement Parameters (ADPs) for the BaSiH_6_ Structure at Ambient Conditions^a^

atom	Wyck	*x*	*y*	*z*	*U*_iso_ (Å^2^)	*B*_iso_ (Å^2^)
Ba	4*e*	0	0.6990(2)^b^	0	0.0198(3)	1.56(2)
Si	4*d*	1/4	1/4	1/2	0.0181(9)	1.43(7)
H1	8*f*	0.8091	0.4674	0.4394	0.038	3
H2	8*f*	0.4384	0.3133	0.5963	0.038	3
H3	8*f*	0.2327	0.0912	0.6531	0.038	3

a Hydrogen atom positions were obtained from DFT relaxation constraint to the experimental lattice, see text. The coordinates and ADPs of the H atoms remained fixed during the refinement.

b Ba is at 4*e* (0, 0.75, 0) for an idealized NaCl-type arrangement of Ba and Si atoms.

**Figure 3:**
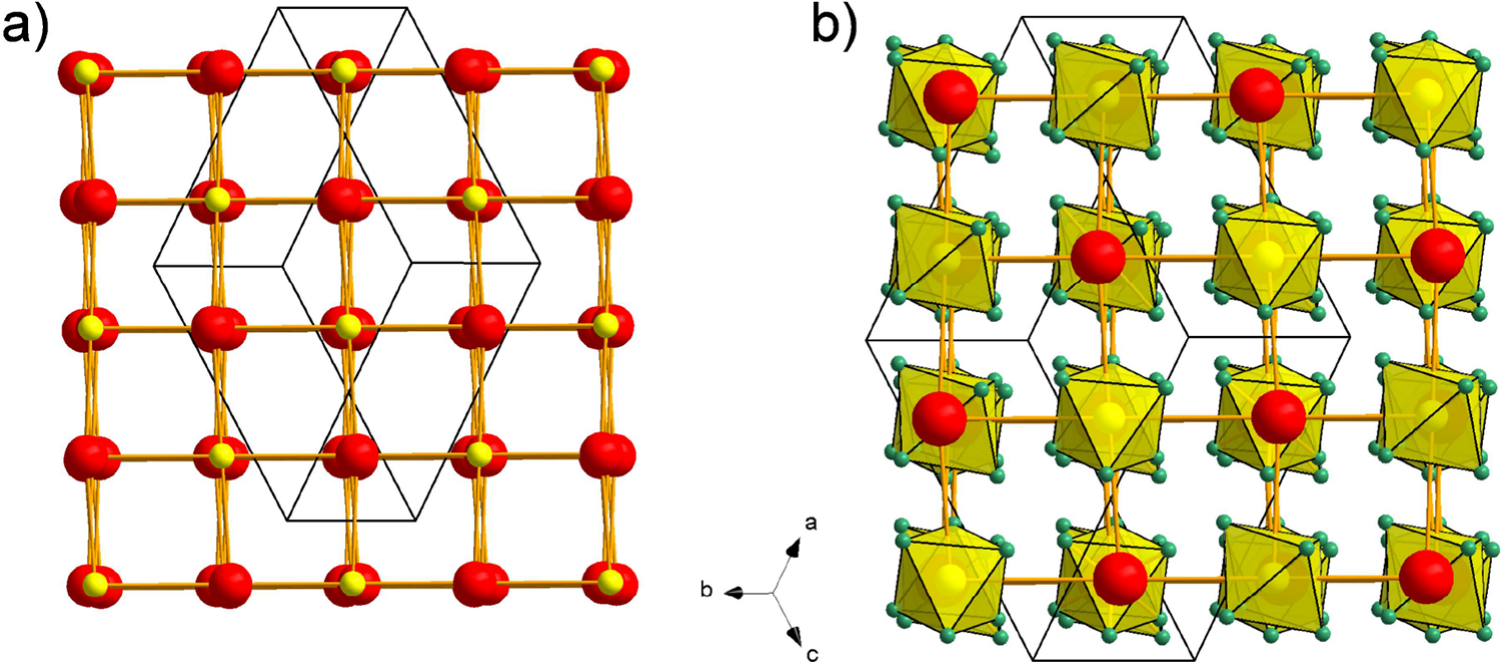
(a) Projection of the monoclinic BaSiH_6_ structure showing a nearly ideal fcc NaCl structure arrangement of Ba and Si atoms (red and yellow spheres, respectively). In (b) the H atoms are added (green spheres).

The monoclinic crystal structure of BaSiH_6_ is built of Ba^2+^ and octahedral complex ions SiH_6_^2–^ which are the characteristic constituents of hydridosilicates. The SiH_6_^2–^ octahedra appear in two orientations, which are related through 2_1_ symmetry axes. They are hosted within irregular octahedra of Ba^2+^ counterions (*d*_Ba–Ba_ = 4.78–5.26 Å) with their corners pointing to triangle faces and edges of the Ba_6_ octahedron (Figure 1). The monoclinic unit cell blurs an almost ideal NaCl-type arrangement of cations and centers of complex anions (Figure 3), which is realized in the fcc structure of NaPF_6_ (with PF_6_^–^ octahedra aligned in the same orientation as the Na_6_ ones)^33^ but from which monoclinic KPF_6_ severely deviates. The parameters of the pseudocubic triclinic unit cell of BaSiH_6_ are in the range 7.01–7.04 Å and 87.2–89.3° (Table 3). Nearest neighbor Ba–Si distances are between 3.35 and 3.70 Å.

**Table 3:** Relevant Interatomic Distances in BaSiH_6_^a^

atom pair			*d* (Å)	atom pair			*d* (Å)
Si	H2	×2	1.590	Ba	Ba	×2	4.775
	H3	×2	1.608		Ba	×2	4.855
	H1	×2	1.609		Ba	×4	4.937
Ba	Si	×2	3.354		Ba	×2	5.123
	Si	×2	3.512		Ba	×2	5.254
	Si	×2	3.695	Ba	H3	×2	2.605
Si	Si	×2	4.855		H3	×2	2.607
	Si	×4	4.937		H2	×2	2.693
	Si	×4	4.996		H2	×2	2.798
	Si	×2	5.099		H1	×2	2.822
					H1	×2	2.892
					H1	×2	2.965
					H2	×2	3.274

a Estimated standard deviations are either smaller than 0.001 Å or not defined (when involving H atoms).

**Figure 4:**
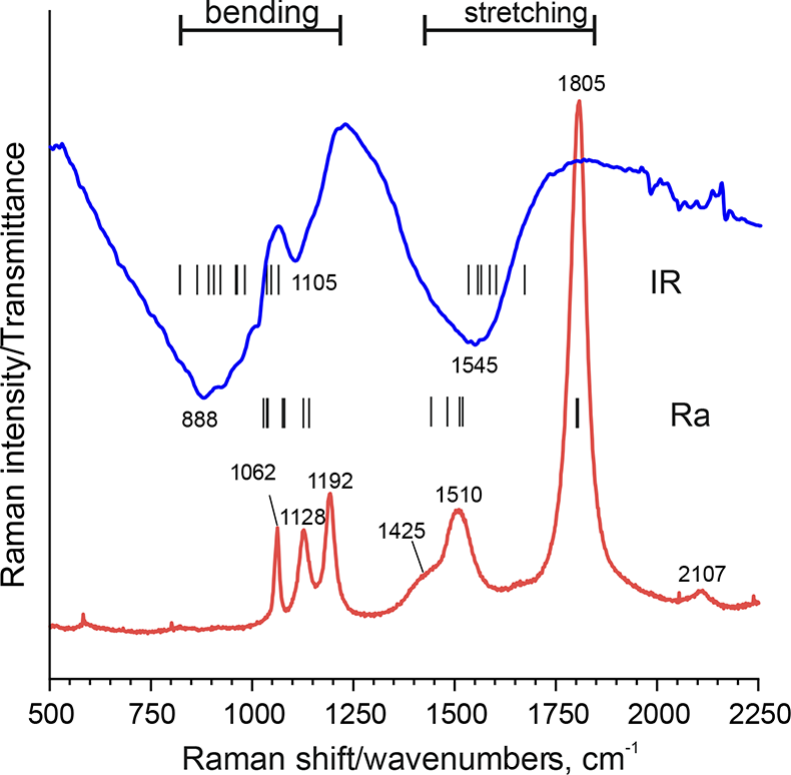
Raman (lower curve) and IR spectrum (upper curve) of BaSiH_6_. The bars represent the position of DFT calculated Si–H stretching and bending modes. The unassigned band at 2107 cm^–1^ in the Raman spectrum may correspond to an overtone. Note that Si–O bands from Ba_2_SiO_4_ orthosilicate impurity are expected to be below 1000 cm^–1^ and will not interfere with BaSiH_6_ Si–H bands in the Raman spectrum.^36^ In contrast, in the IR spectrum, Si–O stretching and Si–O–H bending bands from partial decomposition of the sample may be present.

The local point group symmetry of the SiH_6_^2–^ ion is *C*_i,_ and there are two octahedra in the primitive unit cell. Despite the low symmetry, the Si–H distances are almost equal (*d*_Si–H_ = 1.60–1.62 Å) and angles are close to ideal (89–91°). Consequently, one would expect that the internal modes of SiH_6_^2–^ follow the *O*_h_ pattern of three stretching modes (A_1g_, E_g_, T_1u_) and three bending modes (T_2g_, T_1u_, T_2u_) and that the *O*_h_ selection rules are largely obeyed.^32,34^ The IR and Raman spectra of BaSiH_6_, shown in Figure 4, reveal the location of bending and stretching modes between 800–1250 and 1400–1810 cm^–1^, respectively, which is also seen in the DFT calculated zone center optical modes. The Raman spectrum shows six vibrational bands, three in the stretching and three in the bending mode regions, which implies that the *O*_h_-degenerate modes E_g_ (stretch) and T_1g_ (bend) all appear considerably split by several tens of cm^–1^. The removal of the *O*_h_ degeneracy is not visible in the IR spectrum, which only shows two broad bands, one in the stretching region (accounting for T_1u_) and one in the bending region (accounting for T_1u_ and the *O*_h_-forbidden T_2u_). An additional IR signal may come from Fermi resonance,^33^ but this is not clear. The symmetric stretch is at the highest wavenumber, around 1800 cm^–1^, which relates well to K_2_SiH_6_ and Rb_2_SiH_6_ (which are also built of separated SiH_6_^2–^ octahedra but with local point group symmetry *O*_h_).^1^ The associated Si–H stretching force constant is thus considered similar (∼1.3 N/cm),^35^ confirming that SiH_6_^2–^ is a weakly bonded moiety compared to regular-valent silicon hydride species.^2^

The electronic band structure of BaSiH_6_ shows considerable dispersion of the occupied bands (1–3 eV), as depicted in Figure S7. Yet these bands reflect clearly the molecular orbital energy levels of octahedral SiH_6_^2–^ (i.e., a_1g_, t_1u_, e_g_) which confirms the ionic character of this compound. The highest lying e_g_-type band is nonbonding and primarily composed of H states. The band gap appears to be direct with a size of about 2.5 eV. Typically, DFT calculated band gaps are underestimated when using the PBE functional. Here, however, the reddish appearance of a sintered bulk sample (cf. Figure 2, inset) suggests quite good agreement.

**Figure 5:**
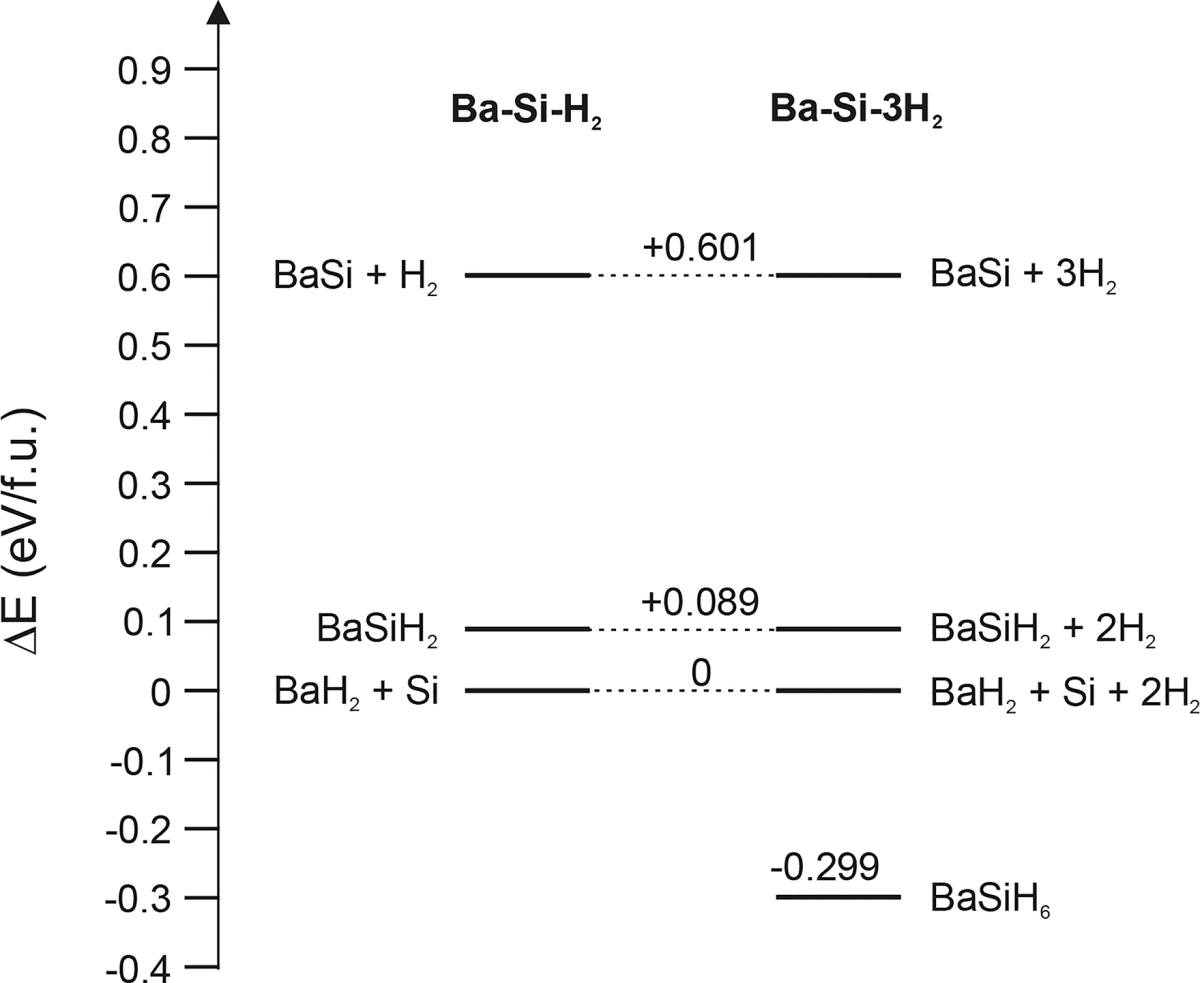
DFT calculated energy differences for the pseudobinary compositions BaSi–H_2_ and BaSi–3H_2_.

Multitemperature PXRD measurements revealed a limited thermal stability of BaSiH_6_. At about 95 °C, decomposition onset to BaH_2_ and Si is observed (Figure S4). Nevertheless, according to DFT calculations, BaSiH_6_ is a thermodynamically stable compound (with respect to the most stable component mixture BaH_2_ + Si + H_2_). In fact, at ambient pressure, BaSiH_6_ represents the only stable ternary compound in the Ba–Si–H system, whereas the (stoichiometric) Zintl phase hydride BaSiH_2_ is not (Figure 5). This follows earlier observations that hydridosilicates appear generally thermodynamically stable at ambient pressure, albeit high pressure is needed for their synthesis for raising thermal stability beyond required reaction temperatures. In this respect, and as mentioned earlier, silicide Zintl phases (or also Zintl phase hydrides) can favorably act as reactive precursors with low kinetic barriers for hydrogenation.

Recently, Lucrezi et al. reported a computational study of the even more hydrogen-rich polyhydride BaSiH_8_.^11^ BaSiH_8_ was predicted to be thermodynamically stable at pressures above 130 GPa. However, minimum pressures for dynamic and kinetic stability (which are the important parameters for retaining a high-pressure material toward ambient pressure) were found to be considerably lower, at around 25 and 30 GPa, respectively.^11,37^ BaSiH_8_ is potentially a promising superconducting material with a critical temperature *T*_C_ around 80 K (rather independent of pressure). The existence of BaSiH_6_ was not known to these authors and also their results were based on zero Kelvin calculations. In light of our findings, it would be interesting and also important to reassess thermodynamic, kinetic, and dynamic stabilities for BaSiH_8,_ considering both the presence of BaSiH_6_ in the ternary system Ba–Si–H and elevated temperatures of >400 °C necessary for inducing reactions.

**Figure 6:**
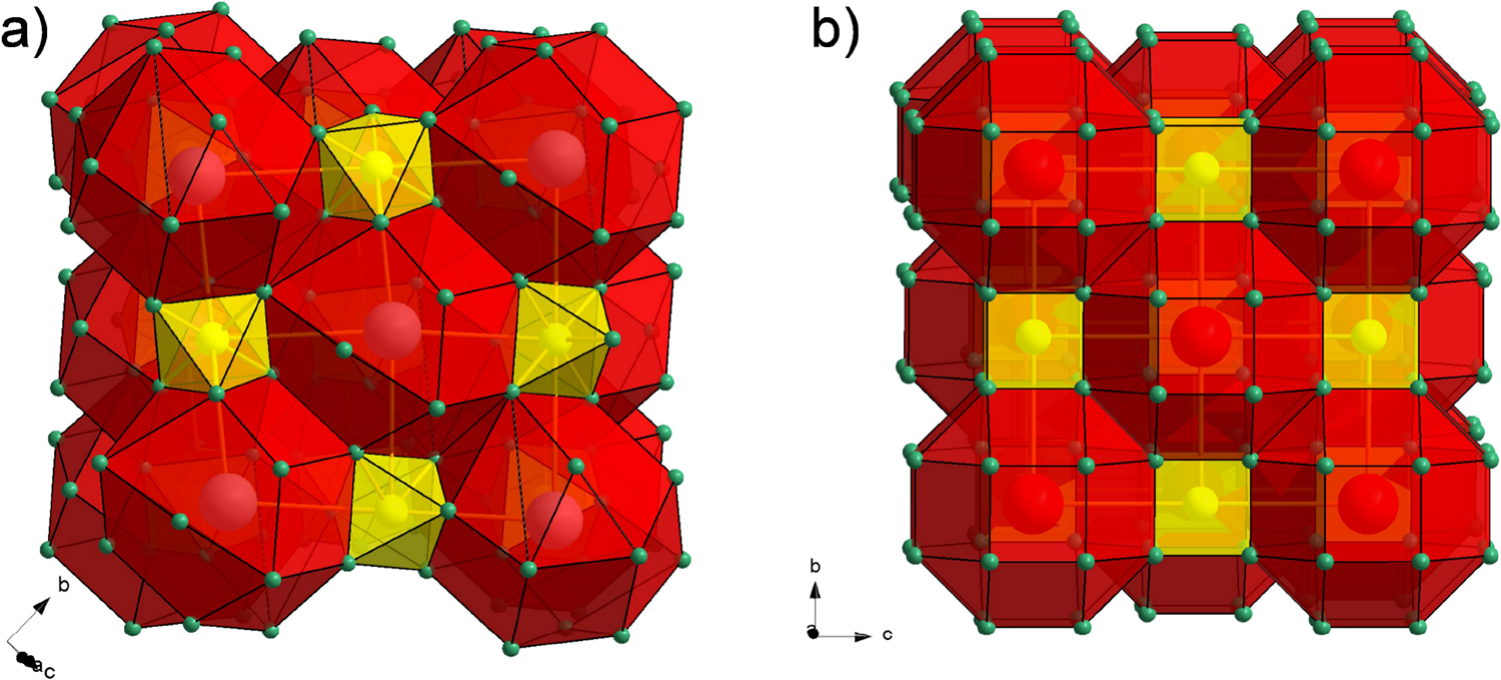
Comparison of the crystal structures of monoclinic BaSiH_6_ (a) and cubic BaSiH_8_ (b). Ba and Si coordination by H are depicted as red and yellow polyhedra, respectively.

Here, we infer that BaSiH_6_ represents a precursor, or intermediate, to the higher hydride BaSiH_8_, potentially accessible at pressures by far exceeding 10 GPa, and motivate this by their close structural relationship. In cubic BaSiH_8_ (space group *Fm*3̅*m*), Ba and Si atoms form an ideal NaCl-type structure arrangement. H atoms are situated on the 32*f* (*x*,*x,x*) position) and surround the Si atoms cubically. Interestingly, the calculated lattice parameter of BaSiH_8_ at ambient pressure (where BaSiH_8_ would be both dynamically and kinetically unstable) is close to 7 Å,^11,37^ implying an almost equal dimension of the NaCl lattice hosting SiH_6_ octahedra in BaSiH_6_ and SiH_8_ cubes in BaSiH_8_ (Figure 6). Obviously, the “insertion” of additional 2 H atoms per formula unit into the BaSiH_6_ structure will change the Ba–H coordination drastically. In BaSiH_8_, Ba is coordinated by 24 H atoms, forming a rhombicuboctahedron at a distance 2.87 Å, whereas in BaSiH_6_, Ba is coordinated rather irregularly by 14 H atoms in a distance range 2.61–2.97 Å.

## Conclusions

The hydridosilicate BaSiH_6_ can be synthesized when reacting the Zintl phase hydride BaSiH_2–*x*_ with H_2_ fluid at pressures above 4 GPa and subsequent decompression to ambient pressure. The monoclinic crystal structure consists of Ba^2+^ and hypervalent complex SiH_6_^2–^ ions. Their mutual arrangement corresponds closely to an ideal fcc NaCl structure. BaSiH_6_ is the first hydridosilicate with an alkaline earth metal and its discovery suggests a broader compositional variability in the cationic constituents of this relatively new class of compounds. Like the previously described alkali metal hydridosilicates, BaSiH_6_ is a thermodynamically stable compound at ambient pressure, albeit with a rather low thermal stability. Its synthesis via BaSiH_2–*x*_ can be upscaled which eventually, with pure samples, will allow for detailed physical property characterization/measurements, including heat capacity, dynamic properties from inelastic/quasielastic neutron scattering spectroscopy, semiconductor properties, and H^–^ ion conductivity (probably only seen at pressure upon increased thermal stability). The structural properties of BaSiH_6_ suggest that it represents a bridge to the predicted superconducting polyhydride BaSiH_8_^11^ which can be expected from hydrogenations at ultrahigh pressures of about 100 GPa and for which BaSiH_6_ may serve as a most suitable precursor.

## Supporting Information Available

Detailed description of synthesis and sample preparations; details of the PXRD characterization of products; detailed description of the high-pressure neutron diffraction experiment and the refinement of neutron diffraction data; structure parameters for BaSiD_6_ at 2.5 GPa; detailed description of the multitemperature synchrotron powder diffraction experiment and evaluation of data; DFT relaxed structure parameters of for *C*2/*c* BaSiH_6_; and electronic band structure and density of states for *C*2/*c* BaSiH_6_ (PDF)

## Accession Codes

Deposition Numbers 2420757 and 2420758 contain the supplementary crystallographic data for this paper. These data can be obtained free of charge via the joint Cambridge Crystallographic Data Centre (CCDC) and Fachinformationszentrum Karlsruhe Access Structures service.

## References

[ref1] Puhakainen K.; Benson D.; Nylén J.; Konar S.; Stoyanov E.; Leinenweber K.; Häussermann U. Hypervalent Octahedral SiH_6_^2–^ Species from High-Pressure Synthesis. Angew. Chem., Int. Ed. 2012, 51 (13), 3156–3160. 10.1002/anie.201108713. 22323241

[ref2] Vekilova O. Yu.; Beyer D. C.; Bhat S.; Farla R.; Baran V.; Simak S. I.; Kohlmann H.; Häussermann U.; Spektor K. Formation and Polymorphism of Semiconducting K_2_SiH_6_ and Strategy for Metallization. Inorg. Chem. 2023, 62 (21), 8093–8100. 10.1021/acs.inorgchem.2c04370. 37188333 PMC10231339

[ref3] Spektor K.; Kohlmann H.; Druzhbin D.; Crichton W. A.; Bhat S.; Simak S. I.; Vekilova O. Y.; Häussermann U. Hypervalent Hydridosilicate in the Na–Si–H System. Front. Chem. 2023, 11, 1251774 10.3389/fchem.2023.1251774. 37744059 PMC10515085

[ref4] Liang T.; Zhang Z.; Feng X.; Jia H.; Pickard C. J.; Redfern S. A. T.; Duan D. Ternary Hypervalent Silicon Hydrides via Lithium at High Pressure. Phys. Rev. Mater. 2020, 4 (11), 113607 10.1103/PhysRevMaterials.4.113607.

[ref5] Liang T.; Zhang Z.; Yu H.; Cui T.; Feng X.; Pickard C. J.; Duan D.; Redfern S. A. T. Pressure-Induced Superionicity of H^–^ in Hypervalent Sodium Silicon Hydrides. J. Phys. Chem. Lett. 2021, 12 (30), 7166–7172. 10.1021/acs.jpclett.1c01809. 34297555

[ref6] Wu S.; Li B.; Chen Z.; Hou Y.; Bai Y.; Hao X.; Yang Y.; Liu S.; Cheng J.; Shi Z. Phase Transitions and Superconductivity in Ternary Hydride Li_2_SiH_6_ at High Pressures. J. Appl. Phys. 2022, 131 (6), 065901 10.1063/5.0080880.

[ref7] Saitoh H.; Machida A.; Aoki K. Synchrotron X-Ray Diffraction Techniques for in Situ Measurement of Hydride Formation under Several Gigapascals of Hydrogen Pressure. Chin. Sci. Bull. 2014, 59 (36), 5290–5301. 10.1007/s11434-014-0543-8.

[ref8] Spektor K.; Crichton W. A.; Filippov S.; Klarbring J.; Simak S. I.; Fischer A.; Häussermann U. Na–Ni–H Phase Formation at High Pressures and High Temperatures: Hydrido Complexes [NiH_5_]^3–^*Versus* the Perovskite NaNiH_3_. ACS Omega 2020, 5 (15), 8730–8743. 10.1021/acsomega.0c00239. 32337435 PMC7178781

[ref9] Spektor K.; Crichton W. A.; Filippov S.; Simak S. I.; Fischer A.; Häussermann U. Na_3_FeH_7_ and Na_3_CoH_6_: Hydrogen-Rich First-Row Transition Metal Hydrides from High Pressure Synthesis. Inorg. Chem. 2020, 59 (22), 16467–16473. 10.1021/acs.inorgchem.0c02294. 33141575 PMC7672699

[ref10] Nylén J.; Sato T.; Soignard E.; Yarger J. L.; Stoyanov E.; Häussermann U. Thermal Decomposition of Ammonia Borane at High Pressures. J. Chem. Phys. 2009, 131 (10), 104506 10.1063/1.3230973.

[ref11] Lucrezi R.; Di Cataldo S.; von der Linden W.; Boeri L.; Heil C. In-Silico Synthesis of Lowest-Pressure High-*T*_c_ Ternary Superhydrides. npj Comput. Mater. 2022, 8, 119 10.1038/s41524-022-00801-y.

[ref12] Auer H.; Guehne R.; Bertmer M.; Weber S.; Wenderoth P.; Hansen T. C.; Haase J.; Kohlmann H. Hydrides of Alkaline Earth–Tetrel (AeTt) Zintl Phases: Covalent Tt–H Bonds from Silicon to Tin. Inorg. Chem. 2017, 56 (3), 1061–1071. 10.1021/acs.inorgchem.6b01944. 28098994

[ref13] Stoyanov E.; Häussermann U.; Leinenweber K. Large-Volume Multianvil Cells Designed for Chemical Synthesis at High Pressures. High Pressure Res. 2010, 30 (1), 175–189. 10.1080/08957950903422444.

[ref14] Farla R.; Bhat S.; Sonntag S.; Chanyshev A.; Ma S.; Ishii T.; Liu Z.; Néri A.; Nishiyama N.; Faria G. A.; Wroblewski T.; Schulte-Schrepping H.; Drube W.; Seeck O.; Katsura T. Extreme Conditions Research Using the Large-Volume Press at the P61B Endstation, PETRA III. J. Synchrotron Rad. 2022, 29 (2), 409–423. 10.1107/S1600577522001047. PMC8900846 35254304

[ref15] Hattori T.; Sano-Furukawa A.; Arima H.; Komatsu K.; Yamada A.; Inamura Y.; Nakatani T.; Seto Y.; Nagai T.; Utsumi W.; Iitaka T.; Kagi H.; Katayama Y.; Inoue T.; Otomo T.; Suzuya K.; Kamiyama T.; Arai M.; Yagi T. Design and Performance of High-Pressure PLANET Beamline at Pulsed Neutron Source at J-PARC. Nucl. Instrum. Methods Phys. Res., Sect. A 2015, 780, 55–67. 10.1016/j.nima.2015.01.059.

[ref16] Dippel A.-C.; Liermann H.-P.; Delitz J. T.; Walter P.; Schulte-Schrepping H.; Seeck O. H.; Franz H. Beamline P02.1 at PETRA III for High-Resolution and High-Energy Powder Diffraction. J. Synchrotron Rad. 2015, 22 (3), 675–687. 10.1107/S1600577515002222. PMC4416682 25931084

[ref17] Shirley R. Crysfire 2004: An interactive powder indexing support system; Crysfire: Surrey, UK, 2004.

[ref18] Petříček V.; Dušek M.; Palatinus L. Crystallographic Computing System JANA2006: General Features. Z. Kristallogr. - Cryst. Mater. 2014, 229 (5), 345–352. 10.1515/zkri-2014-1737.

[ref19] Oganov A. R.; Glass C. W. Crystal Structure Prediction Using Ab Initio Evolutionary Techniques: Principles and Applications. J. Chem. Phys. 2006, 124 (24), 244704 10.1063/1.2210932. 16821993

[ref20] Lyakhov A. O.; Oganov A. R.; Stokes H. T.; Zhu Q. New Developments in Evolutionary Structure Prediction Algorithm USPEX. Comput. Phys. Commun. 2013, 184 (4), 1172–1182. 10.1016/j.cpc.2012.12.009.

[ref21] Oganov A. R.; Lyakhov A. O.; Valle M. How Evolutionary Crystal Structure Prediction Works—and Why. Acc. Chem. Res. 2011, 44 (3), 227–237. 10.1021/ar1001318. 21361336

[ref22] Kresse G.; Hafner J. Ab Initio Molecular Dynamics for Liquid Metals. Phys. Rev. B 1993, 47 (1), 558–561. 10.1103/PhysRevB.47.558. 10004490

[ref23] Kresse G.; Furthmüller J. Efficient Iterative Schemes for Ab Initio Total-Energy Calculations Using a Plane-Wave Basis Set. Phys. Rev. B 1996, 54 (16), 11169 10.1103/PhysRevB.54.11169. 9984901

[ref24] Blöchl P. E. Projector Augmented-Wave Method. Phys. Rev. B 1994, 50 (24), 17953–17979. 10.1103/PhysRevB.50.17953. 9976227

[ref25] Hohenberg P.; Kohn W. Inhomogeneous Electron Gas. Phys. Rev. 1964, 136 (3B), B864–B871. 10.1103/PhysRev.136.B864.

[ref26] Perdew J. P.; Burke K.; Ernzerhof M. Generalized Gradient Approximation Made Simple. Phys. Rev. Lett. 1996, 77 (18), 3865–3868. 10.1103/PhysRevLett.77.3865. 10062328

[ref27] Perdew J. P.; Burke K.; Ernzerhof M. Errata: Generalized Gradient Approximation Made Simple [Phys. Rev. Lett. 77, 3865 (1996)]. Phys. Rev. Lett. 1997, 78 (7), 1396 10.1103/PhysRevLett.78.1396. 10062328

[ref28] Monkhorst H. J.; Pack J. D. Special Points for Brillouin-Zone Integrations. Phys. Rev. B 1976, 13 (12), 5188–5192. 10.1103/PhysRevB.13.5188.

[ref29] Togo A.; Tanaka I. First Principles Phonon Calculations in Materials Science. Scr. Mater. 2015, 108, 1–5. 10.1016/j.scriptamat.2015.07.021.

[ref30] Currao A.; Curda J.; Nesper R. Kann man die Arten von Zintl-Anionen steuern?? Variationen über das Thema Si^2–^ im System Sr/Mg/Si. Z. Anorg. Allg. Chem. 1996, 622 (1), 85–94. 10.1002/zaac.19966220113.

[ref31] Cockcroft J. K. Neutron-Scattering Studies of Order-Disorder Transitions in Hexafluoride Salts ABF_6_. Thesis (D.Phil.), University of Oxford, 1985.

[ref32] Parker S. F.; Williams K. P. J.; Smith T.; Ramirez-Cuesta A. J.; Daemen L. L. Vibrational Spectroscopy of Hexahalo Complexes. Inorg. Chem. 2022, 61 (15), 5844–5854. 10.1021/acs.inorgchem.2c00125. 35380803 PMC9171826

[ref33] Kitashita K.; Hagiwara R.; Ito Y.; Tamada O. Crystal Structures of Some Cubic Hexafluorophosphates at Ambient Temperatures. J. Fluorine Chem. 2000, 101 (2), 173–179. 10.1016/S0022-1139(99)00155-4.

[ref34] Parker S. F. Spectroscopy and Bonding in Ternary Metal Hydride Complexes—Potential Hydrogen Storage Media. Coord. Chem. Rev. 2010, 254 (3–4), 215–234. 10.1016/j.ccr.2009.06.016.

[ref35] Kranak V. F.; Lin Y.-C.; Karlsson M.; Mink J.; Norberg S. T.; Häussermann U. Structural and Vibrational Properties of Silyl (SiH_3_^–^) Anions in KSiH_3_ and RbSiH_3_: New Insight into Si–H Interactions. Inorg. Chem. 2015, 54 (5), 2300–2309. 10.1021/ic502931e. 25668724

[ref36] Handke M.; Urban M. IR and Raman Spectra of Alkaline Earth Metals Orthosilicates. J. Mol. Struct. 1982, 79, 353–356. 10.1016/0022-2860(82)85083-7.

[ref37] Lucrezi R.; Kogler E.; Di Cataldo S.; Aichhorn M.; Boeri L.; Heil C. Quantum Lattice Dynamics and Their Importance in Ternary Superhydride Clathrates. Comm 2023, 6, 298 10.1038/s42005-023-01413-8. PMC11549051 39524969

