# Supplementary material for: Synthesis of BaSiH6 Hydridosilicate at High Pressures—A Bridge to BaSiH8 Polyhydride
Source: ACS Omega. 2025 Apr 7;10(15):15029–35. doi: 10.1021/acsomega.4c10502 (PMC12019724; doi:10.1021/acsomega.4c10502)
Supplement: Supplementary file 1 — ao4c10502_si_001.pdf [file ao4c10502_si_001.pdf]

SUPPORTING INFORMATION

Synthesis of $BaSiH_6$ hydridosilicate at high pressures – a bridge to $BaSiH_8$ polyhydride?

Doreen C. Beyer,[1] Kristina Spektor,[2,*] Olga Yu. Vekilova,[3] Jekabs Grins,[3] Paulo H. Barros Brant Carvalho,[4] Logan J. Leinbach,[5] Michael Sannemo-Targama,[3] Shrikant Bhat,[2] Volodymyr Baran,[2] Martin Etter,[2] Asami Sano-Furukawa,[6] Takanori Hattori,[6] Holger Kohlmann,[1] Sergei I. Simak,[7,8] Ulrich Häussermann[3,*]

[1]*Leipzig University, Institute for Inorganic Chemistry and Crystallography, Johannisallee 29, D-04103 Leipzig, Germany*

[2]*Deutsches Elektronen-Synchrotron DESY, Notkestraße 85, D-22607 Hamburg, Germany*

[3]*Department of Materials and Environmental Chemistry, Stockholm University, SE-10691 Stockholm, Sweden*

[4]*Department of Chemistry – Ångström, Uppsala University, SE-75121 Uppsala, Sweden*

[5]*Eyring Materials Center, Arizona State University, Tempe, AZ* 85287*, USA*

[6]*J-PARC Center, Japan Atomic Energy Agency, 2-4 Shirakata, Tokai-mura, Naka-gun, Ibaraki 319-1195, Japan*

[7]*Theoretical Physics Division, Department of Physics, Chemistry and Biology (IFM) Linköping University, SE-581 83, Linköping, Sweden*

[8]*Department of Physics and Astronomy, Uppsala University, SE-75120 Uppsala, Sweden*

Table of contents

Synthesis and sample preparation

- Figure S1. PXRD patterns for $BaSiH_{2-x}$ and $BaSiD_{2-x}$ precursors.

PXRD characterization of $BaSiH_6$ products

- Figure S2. Rietveld fit of the $BaSiH_6$ structure to a synchrotron PXRD pattern of a bulk sample produced at ASU.

High pressure neutron powder diffraction experiment

- Figure S3. Rietveld fit of the $BaSiH_6$ structure to *in situ* neutron diffraction data at 2.5 GPa and room temperature.

- Table S1. Crystallographic data for $BaSiD_6$ at 2.5 GPa and room temperature.

Multi-temperature SC-PXRD experiment

- Figure S4. Compilation of synchrotron PXRD patterns of $BaSiH_6$ upon heating.

- Figure S5. Sample temperature as a function of time during the multi-temperature PXRD experiment.

- Figure S6. Evolution of lattice parameters a (a), b (b), c (c), β (d) and unit cell volume V (e) of $BaSiH_6$ as a function of temperature.

Calculations

- Table S2. Fractional coordinates for $BaSiH_6$ from unconstrained DFT relaxation.

- Figure S7. Electronic band structure and density of states for $BaSiH_6$.

Synthesis and sample preparation

Synthesis of $BaSiD_{2-x}$ precursor: $BaSiD_{2-x}$ (x approx. 0.15) were prepared analogous to $BaSiH_{2-x}$ by sintering BaSi in a deuterium atmosphere (D_2, Air Liquide, 99.8% isotope purity) at 90 bar pressure and 180 °C for 24 h, according to ref. 1. Figure S1 shows the diffraction patterns for $BaSiH_{2-x}$ and $BaSiD_{2-x}$ indicating virtually phase pure samples, the latter with possibly a slight (3 wt%) $BaSi_2$ impurity. However, it is suspected that $BaSiH_{2-x}$ contained a significant amount of BaO and/or BaO_2 impurity (amorphous, since it is not recognized in the PXRD pattern), in order to explain the presence of some Ba_2SiO_4 in the $BaSiH_6$ product. Note, that the Ba_2SiO_4 impurity was not seen in in neutron diffraction experiment (for which $BaSiD_{2-x}$ was employed as starting material). Diffraction patterns were collected on a Huber G670 diffractometer employing Cu Kα_1 radiation. The materials were fixed between two Kapton foils with Apiezon grease. Rietveld analysis[2] of the acquired PXRD patterns was performed using TOPAS software (version 5, Bruker AXS).[3]

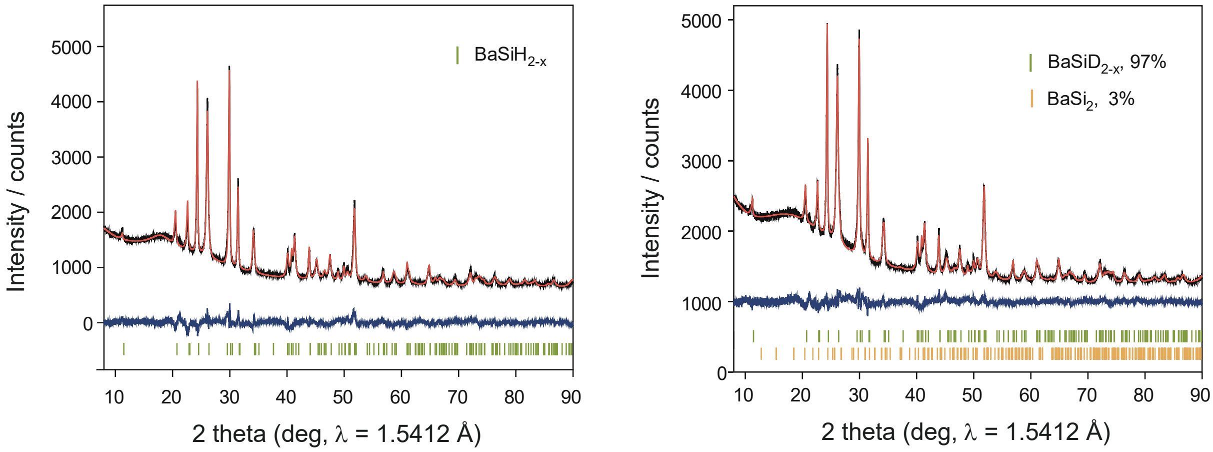

Figure S1. PXRD patterns for $BaSiH_{2-x}$ (left) and $BaSiD_{2-x}$ precursor (right). Black line: measured pattern, red line: calculated pattern, blue line: difference plot.

Synthesis of BD_3ND_3: BD_3ND_3 was used as deuterium source in high pressure neutron diffraction experiments. BD_3ND_3 was obtained by reacting $NaBD_4$ with ammonium formate $NH_4(HCOO)$ which yields NH_3BD_3 and subsequent deuteration of the amine protons with D_2O (according to ref. 4, but instead of three, four cycles of deuteration were performed). NMR analysis (^{11}B, ^{2}H, ^{1}H) revealed an isotope purity of about 85%, with H located on B.

$BaSiH_6$ products obtained from gigapascal hydrogenation of $BaSiH_{2-x}$ contained Ba_2SiO_4 as an impurity phase (3-8 mol%). This impurity was absent in the $BaSiD_6$ sample made from $BaSiD_{2-x}$ despite the similar/nearly identical procedure for both the preparation of $BaSiD_{2-x}$ and $BaSiD_6$. We believe that the $BaSiH_{2-x}$ precursor sample (a batch of about 3 g which was used for all experiments described in this paper) must have contained an impurity involving O (oxide, peroxide, hydroxide) not visible in PXRD. In *in situ* experiments we observed the formation of Ba_2SiO_4 at temperatures between 500 and 550 °C (referring to pressures around 8 GPa), that is, after H_2 release from BH_3NH_3 and after $BaSiH_6$ formation. O-contamination of the $BaSiH_{2-x}$ precursor may have come from BaO/BaO_2 impurity in the Ba used for the synthesis of BaSi which was subsequently used for $BaSiH_{2-x}$ preparation. Or, more likely, due

to an unfortunate (short) exposure of $BaSiH_{2-x}$ to air after its synthesis or during storage (albeit in a glove box) it may have slightly decomposed forming amorphous hydrous products containing Ba and Si.

Sample preparation for high pressure (in situ) diffraction experiments: The sample preparation for high pressure powder X-ray and neutron diffraction experiments, at the facilities PETRA III and MLF/J-PARC, respectively, followed essentially the one for the laboratory synthesis experiments described in the main text. In an Ar-filled glove box powdered precursor was pressed into a pellet and sandwiched between two pellets of H(D)-source, observing a molar ratio $BaSiH(D)_{\sim 1.8}$: $BH(D)_3NH(D)_3$ = 1:1. The three-pellet arrangement was then sealed in a NaCl capsule with dimensions OD/ID 3.0/1.0–1.2 mm, 2.5–3 mm height for the synchrotron experiments and OD/ID 5.5/3.5 mm, 9 mm height for the neutron experiment. The synchrotron experiments utilized the Kawai 6-8 compression geometry with OEL/TEL = 14mm/7mm at P61B, PETRA III, DESY.[5] Assemblies were compressed and heated in the DESY LVP Aster-15 at beamline P61B. Energy-dispersive XRD (ED-XRD) patterns were collected using two germanium solid-state detectors.

PXRD characterization of $BaSiH_6$ products

All of the obtained $BaSiH_6$ products were recovered and handled in an Ar-filled glovebox due to extreme moisture- and air-sensitivity. Recovered $BaSiH_6$ sample pellets produced from *in situ* experiments at P61B, DESY, were typically broken into several pieces, and approximately half of each sample was then sealed (without grinding) inside a 1.0 mm diameter glass capillary. PXRD patterns were collected at the beamline P02.1, PETRA III, DESY[6] using monochromatic synchrotron radiation ($E \approx 60$ keV) at ambient conditions. The samples continuously spun during the data acquisition. The data were recorded using Varex XRD 4343CT flat panel detector (150×150 μm^2 pixel size, 2880 × 2880 pixel area). LaB_6 standard (NIST 660c) sealed in a glass capillary was used for calibration. The measurement shown in Fig. 2 of the main text was acquired with 120 sec exposure time (λ = 0.20734 Å), while the detector was situated at approx. 1300 mm distance from the sample. For multi-temperature measurements at P02.1 the sample produced at ASU was finely ground and sealed in a 0.3 mm diameter fused silica capillary. Capillaries were heated with a mini hot-air blower, which is part of the sample environment at P02.1. Calibration of the sample temperature was performed in a range 30–750 °C by inserting a thermocouple (TC) inside an empty capillary so that TC junction is located approximately at a spot where the beam would hit the sample. During the multi-temperature PXRD experiment the sample was heated in 5–10 °C intervals at 10 °C/min rate followed by 1 min equilibration time. Afterwards, PXRD patterns (λ = 0.20738 Å, sample-to-detector distance ≈ 2100 mm (with the detector corner situated at the beam center)) were acquired at 300 s exposure time, each preceded by a dark image acquisition with 150 sec exposure. The heating was controlled by setting the T on the mini hot-air blower, and actual temperatures shown in Figures S4, S5 were derived later from the calibration. The azimuthal integration of measured 2D patterns was performed using pyFAI software.[7]

Initial indexing of the $BaSiH_6$ peaks from the PXRD patterns of the samples produced at DESY was performed using the DICVOL algorithm included in the Crysfire suite.[8] At first, a smaller monoclinic cell with $Z = 2$ was found ($a \approx 8.74$ Å, $b \approx 4.85$ Å, $c \approx 5.11$ Å, $\beta \approx 126.7$ °, $V \approx 173.8$ Å^3) which featured distorted NaCl-like arrangement of Ba and Si atoms. The correct unit cell was challenging to identify due to significant peak overlap and the presence of the side phase in the product. Later, computational crystal structure predictions suggested a related monoclinic $BaSiH_6$ structure with $C2/c$ (№15) space group. The structure featured the same type of Ba/Si arrangement as the one initially found, but possessed a unit cell with a doubled volume ($Z = 4$). Following this finding, indexing was revisited and the $C2/c$ space group was confirmed from the observed PXRD data. Afterwards, Ba_2SiO_4 was identified as a side product from the residual observed reflections.

Le Bail analysis[9] and Rietveld refinement[2] of the $BaSiH_6$ models against the PXRD data were performed in Jana2006.[10] Prior to the analysis all the data were corrected by subtracting the minimal value of observed intensity from the entire I_{obs} column. The refined parameters included 10th degree polynomial background (in combination with manually assigned points), unit cell dimensions, peak profile parameters (corresponding to pseudo-Voigt function), scale factors, y(Ba) and ADPs for Ba and Si atoms. The CSP-found structure was used as a first

starting model for the refinements. Later, the refinements were finalized using hydrogen positions obtained from a constrained DFT relaxation (see main text and Table 2 for the refinement results). The atomic coordinates and ADPs of the hydrogen atoms remained fixed during the refinements. The U_{iso} (H) were assigned an arbitrary value of 0.038 Å^2 (B_{iso} ≈ 3.0 Å^2). Prior to the plotting and refinements multi-temperature PXRD data were normalized by equalizing averaged I_{obs} from a low 2θ angle region void of peaks (1.60–1.80 °). Ba_2SiO_4 phase (*Pmcn*, *a* ≈ 5.810 Å, *b* ≈ 10.206 Å, *c* ≈ 7.510 Å) was included in all refinements, and cubic Si ($Fd\bar{3}m$, *a* ≈ 5.431 Å) was added to those for multi-temperature data. If present, NaCl peaks (from the capsule material) were added to the excluded regions.

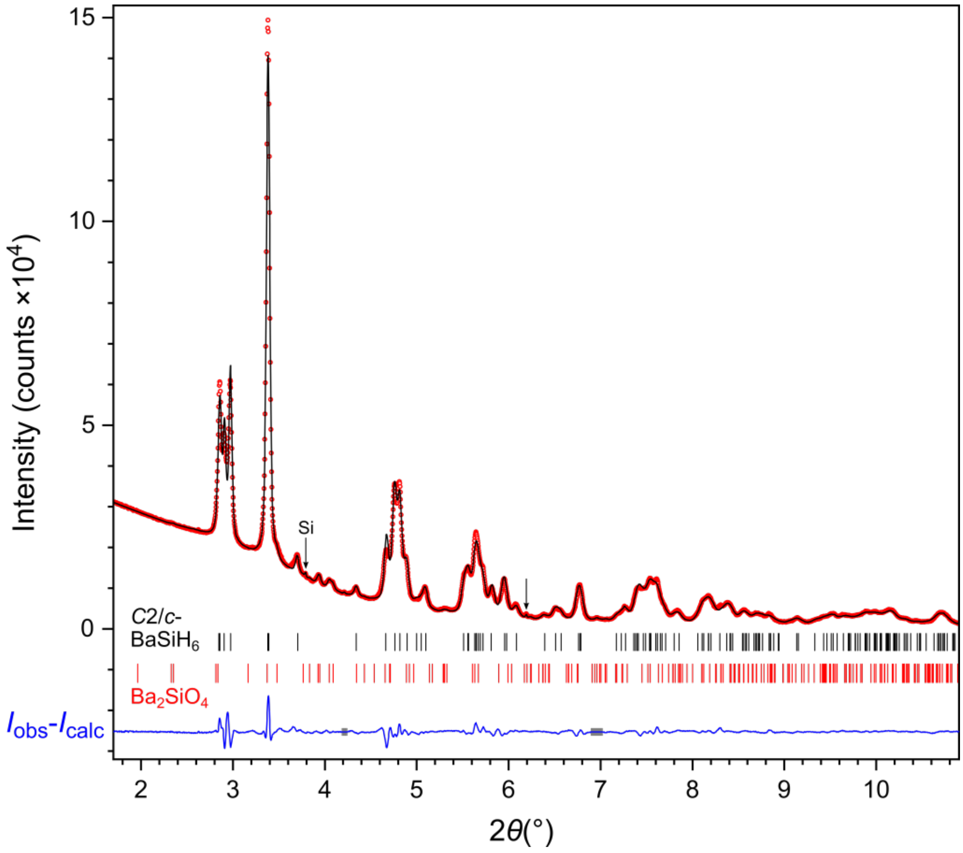

Figure S2. Rietveld fit of the $BaSiH_6$ structure to a synchrotron PXRD pattern (λ = 0.20738 Å) collected at ~30 °C of a bulk sample produced at Arizona State University at ~7 GPa and 550 °C. Cell parameters of $BaSiH_6$ correspond to: *a* = 8.5929(4) Å; *b* = 4.8635(2) Å; *c* = 8.7374(4) Å; β = 108.067(4) °, and *y*(Ba) = 0.7000(2) (R_{obs}=1.64%). Vertical lines are reflection markers for $BaSiH_6$ and Ba_2SiO_4 impurity, Si peaks are marked by black arrows. Excluded regions contain peaks of NaCl (capsule material). Phase fractions correspond to 95.6(1) wt.% (~95.2 mol%) of $BaSiH_6$, 0.5(1) wt.% (~3 mol.%) of Si and 3.9(1) wt.% (~1.8 mol.%) Ba_2SiO_4. Broader/less defined peaks compared to the pattern shown in Fig. 2 in the main text are likely due to fine grinding of the sample. The presence of Si indicates that the synthesis temperature was very close to the decomposition temperature of $BaSiH_6$.

High pressure neutron powder diffraction experiments

Time-of-flight (TOF) neutron diffraction (NPD) experiments were performed at the high-pressure beamline PLANET in the Materials and Life Science Experimental Facility (MLF) at J-PARC.[11] The 6-6 compression geometry with a 15 mm-edge cube and TEL10 mm anvil was utilized.[12] The compression and heating was conducted with the six-axis ATSUHIME press.[13] The sample was compressed to 5.4 GPa and subsequently heated to 400 °C where the onset of deuteration was observed. In order to accelerate the deuteration reaction, the temperature was increased to 500 °C upon which the formation of hexagonal BaD_2 (i.e. the high pressure form of orthorhombic *Pnma* BaH_2)[14] and $Ba_{8-x}Si_{46}$ clathrate was observed. This suggested that at 5.4 GPa and 500 °C $BaSiD_6$ is actually not stable (note that at ambient pressure $BaSiH_6$ decomposes into BaH_2 and Si at around 95 °C, see below) and that the completion of the deuteration reaction should have been performed at a lower temperature. The sample was then cooled to room temperature and the pressure reduced to 2.5 GPa. At this point diffraction data were collected for 24 h for the *d*-range 0.2 – 4.2 Å. During the experiment the sample temperature was controlled based on the heater power, which was based on a pre-calibrated power-temperature relationship. The sample pressure was estimated from the lattice parameter of NaCl (sample container) based on the EOSs by Brown (1999)[15] or Matsui et al (2012).[16]

Data for an empty NaCl capsule inside the graphite heater and pressure medium (approximating the sample geometry under high pressure, including anvil positions and gap), and a vanadium rod within the NaCl capsule were obtained from a previous experiment. Background intensities from the sample container were subtracted from the scattering data, and the sample data were normalized with respect to vanadium. Vanadium data was corrected for absorption, and sample absorption was accounted for during the Rietveld refinement.

The structure of $BaSiD_6$ was refined by the Rietveld method and using the FullProf software.[17] The structure model established from PXRD and DFT calculations (cf. Table 1 and 2 in the main text) was employed. In addition to $BaSiD_6$, the sample was found to contain 11(1) wt% $Pm\overline{3}n$ $Ba_{8-x}Si_{46}$, 5(1) wt% $Fm\overline{3}m$ NaCl and 38(2) wt% $P6_3/mmc$ BaD_2, with a molar ratio clathrate:BaD_2 of roughly 1:1. Data down to 0.70 Å were used, containing 461 reflections from $BaSiD_6$. Deuterium atom sites were assumed to be populated by 86% D and 14% H (reflecting the isotope purity of BD_3ND_3 from NMR analysis). The background was approximated by 19 background points and the pseudo-Voigt peak shape function number 9 was used. An exponential absorption correction A = 0.45 was applied. Refined parameters included phase fractions, lattice parameters, atomic coordinates, and isotropic thermal parameters, with B_{iso}(Ba) = B_{iso}(Si) and B_{iso} equal for all D atoms. The final refinement yielded $\chi^2 = 4.0$ and R_F = 6.7% for $BaSiD_6$. The determined Si–D distances are: Si–D1 = 1.51(1) Å, Si–D2 = 1.49(1) Å, Si–D3 = 1.53(2) Å, with a mean of 1.51 Å.

Table S1. Crystallographic data for $BaSiD_6$ at 2.5 GPa and room temperature from TOF NPD data; space group *C*2/*c* (15), $Z = 4$, $a = 8.427(2)$ Å, $b = 4.767(1)$ Å, $c = 8.502(1)$ Å, $\beta = 108.28(2)$ °, $V = 324.3(1)$ $Å^3$.

**atom**	***Wyck***	***x***	***y***	***z***	B_{iso} ($Å^2$)
Ba	4*e*	0	0.688(2)	0	0.4(1)
Si	4*d*	1/4	1/4	0.5	0.4(1)
D1	8*f*	0.205(1)	0.537(3)	0.548(2)	5.7(2)
D2	8*f*	0.432(1)	0.296(3)	0.585(2)	5.7(2)
D3	8*f*	0.236(2)	0.105(2)	0.655(2)	5.7(2)

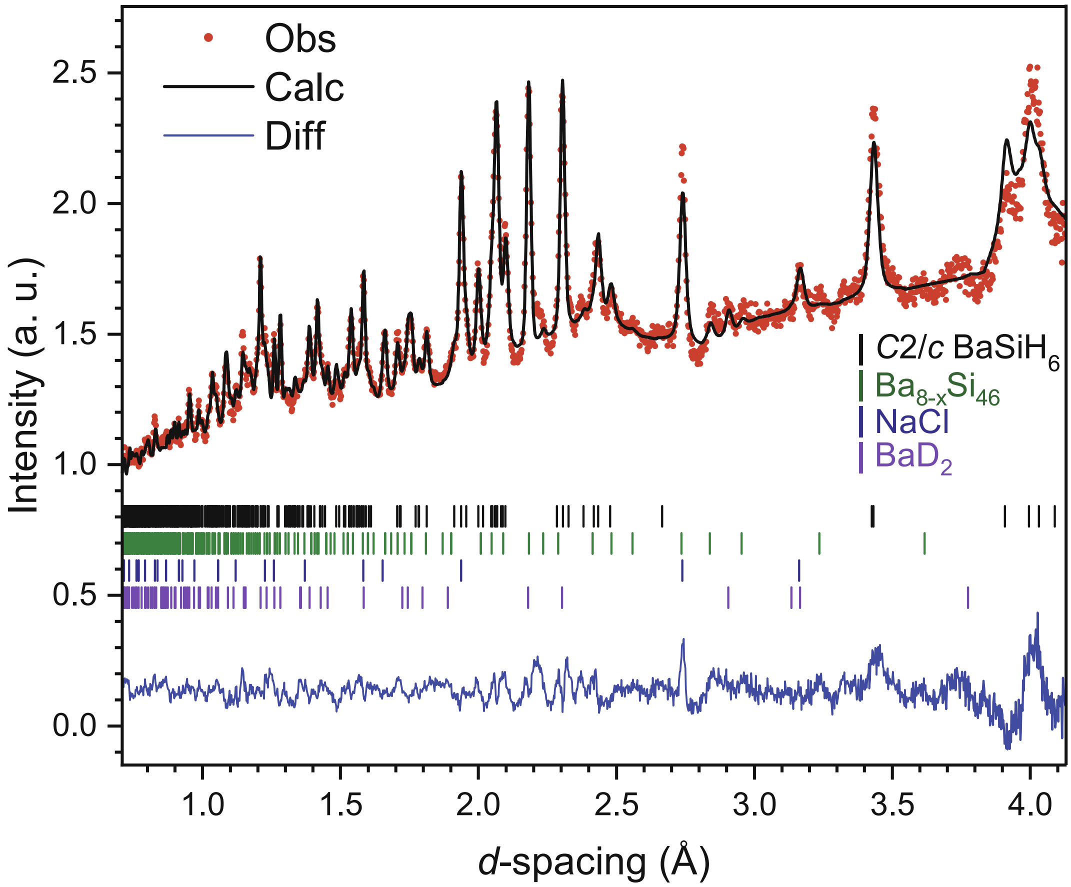

Figure S3. Rietveld fit to NPD data collected at 2.5 GPa and room temperature. Reflection markers are, from top to bottom, for *C*2/*c* $BaSiD_6$, *Pm*$\overline{3}$*n* $Ba_{8-x}Si_{46}$, *Fm*$\overline{3}$*m* NaCl and *P*6_3/*mmc* BaD_2.

Multi-temperature SC-PXRD

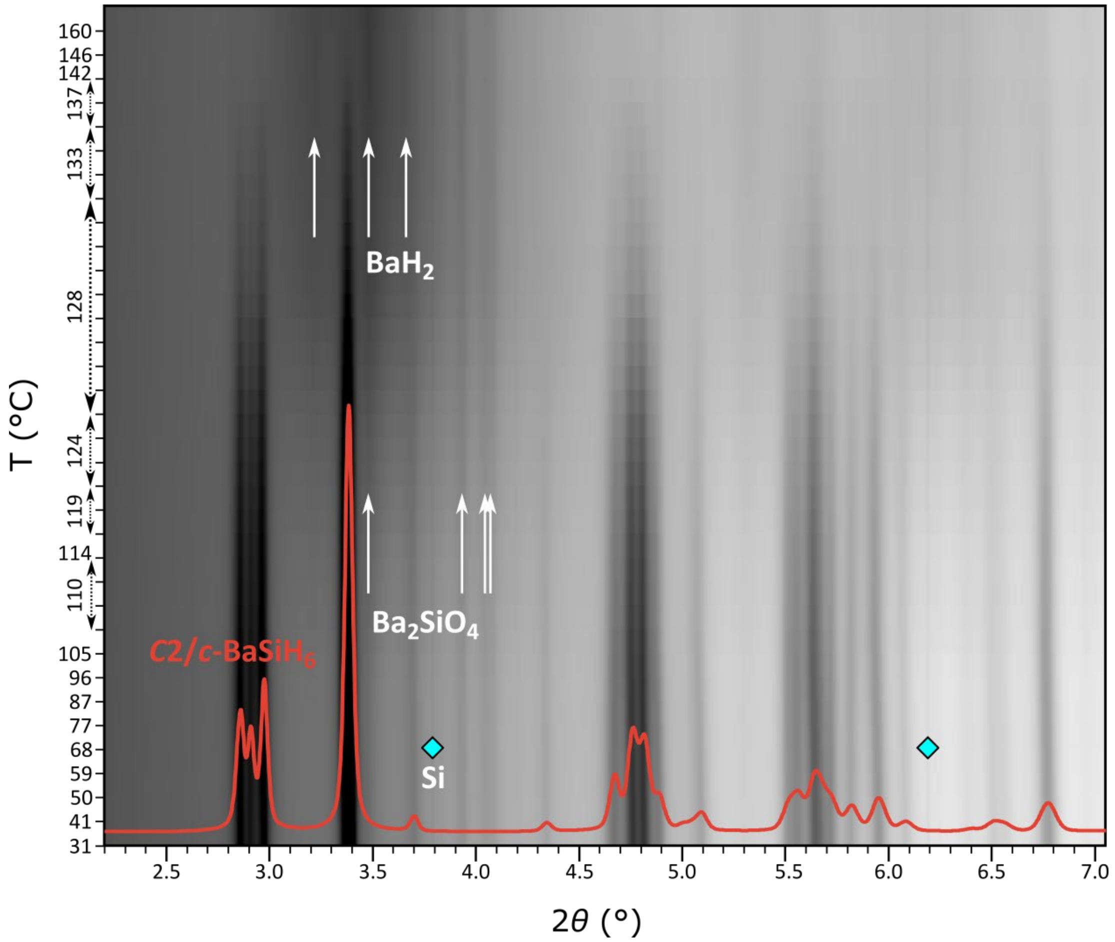

Figure S4. Compilation of synchrotron powder X-ray diffraction patterns (λ = 0.20738 Å) of $BaSiH_6$ (with Ba_2SiO_4 and Si impurities) upon heating. Decomposition into BaH_2 and Si is seen above 95 °C. The plot was produced with the Fit2D software.[18]

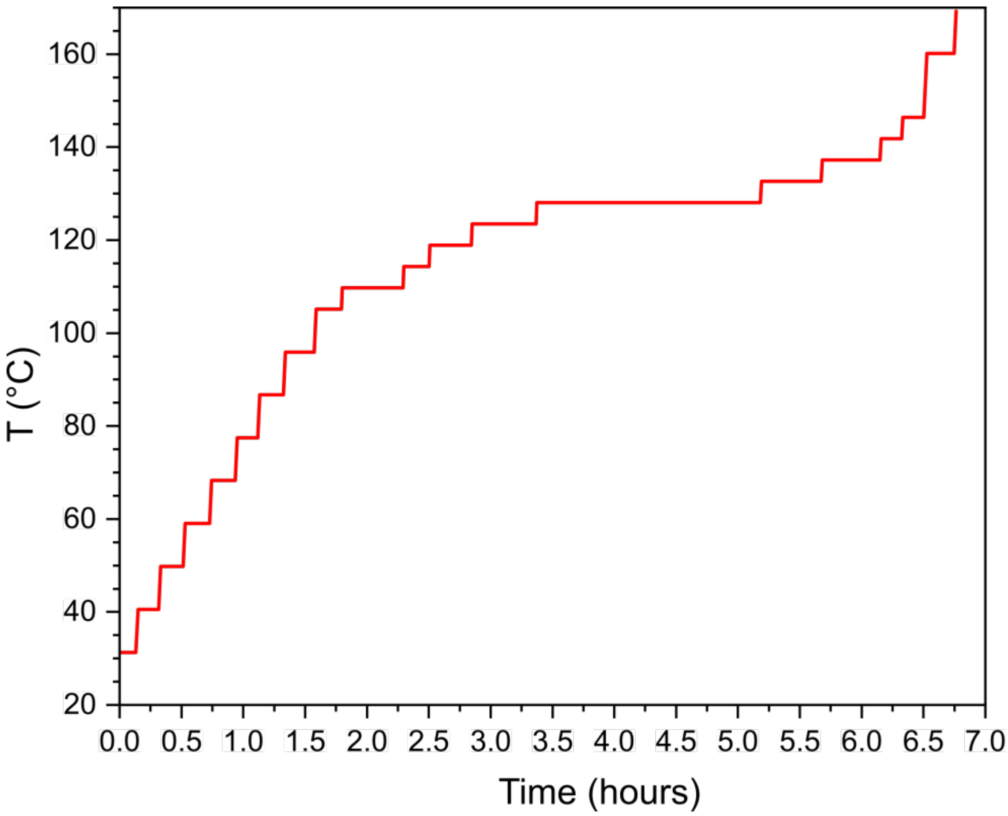

Figure S5. Sample temperature as a function of time during the multi-temperature PXRD experiment at P02.1 (see Figure S4). PXRD patterns were acquired exclusively during temperature dwells. While heating to 170 °C the capillary broke, due to hydrogen buildup accompanying $BaSiH_6$ decomposition, hence no patterns were collected past this point. The event was followed by immediate cooling back to room temperature.

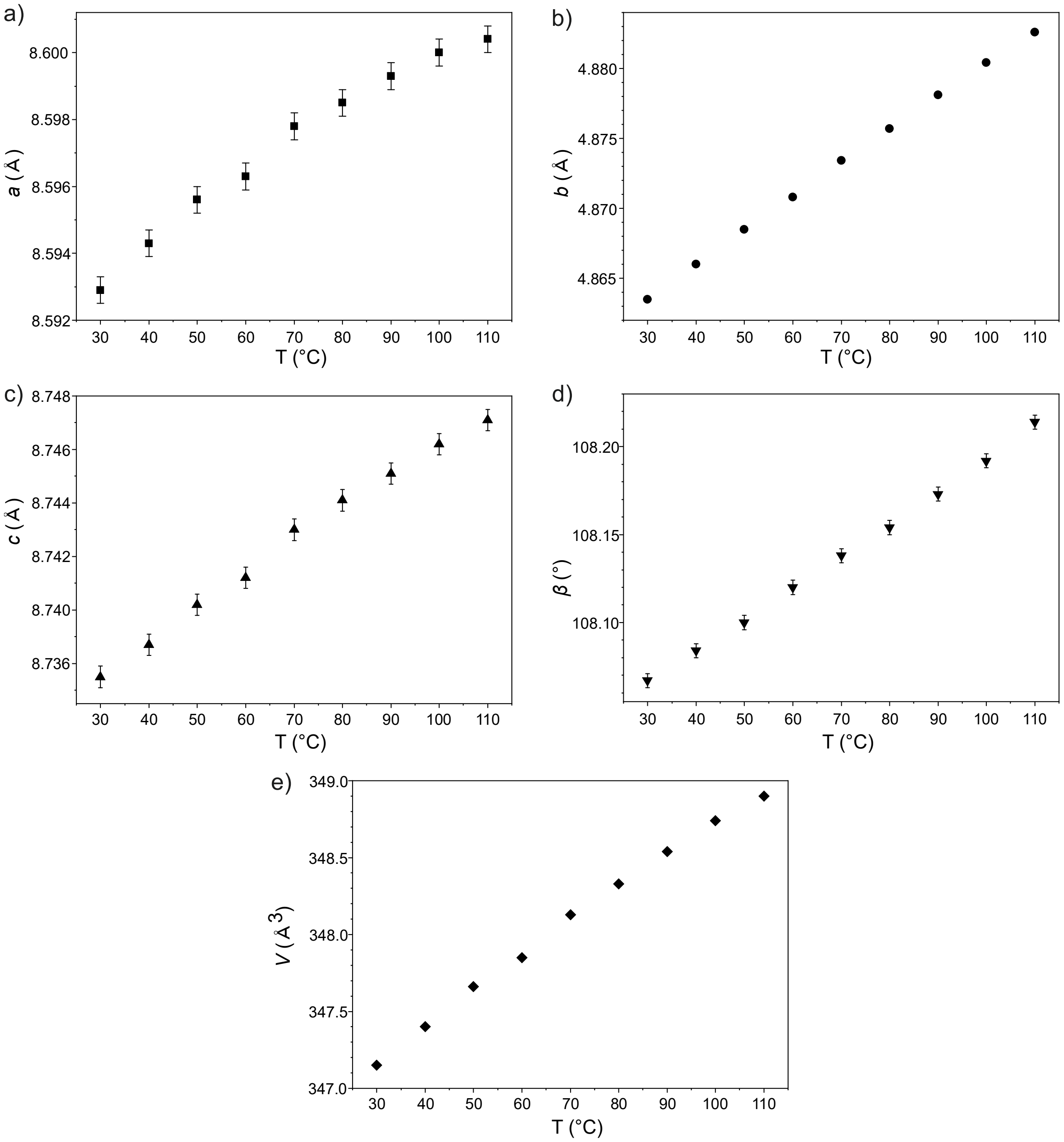

Figure S6. Evolution of lattice parameters *a* (a), *b* (b), *c* (c), *β* (d) and unit cell volume *V* (e) of $BaSiH_6$ as a function of temperature. Errors are not shown in the plots if smaller than the symbol sizes.

Calculations

Table S2. Fractional coordinates for *C*2/*c* $BaSiH_6$ from unconstrained DFT relaxation, producing the unit cell parameters *a* = 8.8778 Å, *b* = 4.7719 Å, *c* = 8.8541 Å, *β* = 104.27 ° (*V* = 363.5 Å^3)

**Atom**	***Wyck***	***x***	***y***	***z***
Ba	4*e*	0	0.6502	0
Si	4*d*	1/4	1/4	1/2
H1	8*f*	0.8326	0.4736	0.4540
H2	8*f*	0.4174	0.3491	0.6029
H3	8*f*	0.2223	0.0879	0.6489

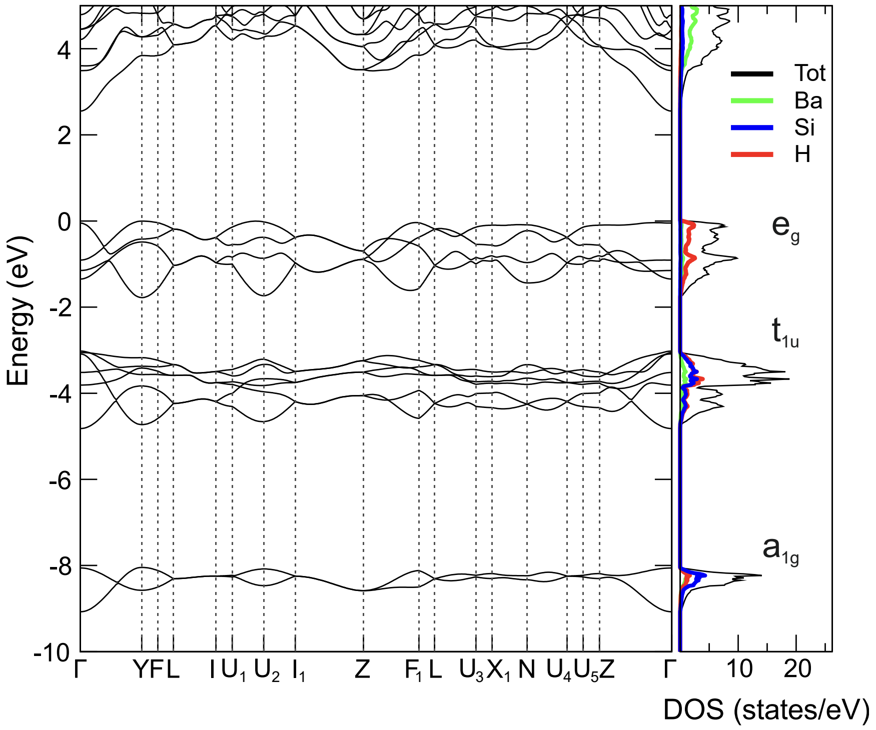

Figure S7. Electronic band structure and density of states (DOS) for $BaSiH_6$, referring to the completely DFT relaxed structure (cf. Table S2). Occupied bands are labelled according to the O_h molecular orbital energy levels of SiH_6^{2-}. The a_{1g} and t_{1u} type bands are Si-H bonding whereas the e_g type band is non-bonding. The Wigner-Seitz radii of spheres for the partial DOS calculations were 1.98 Å, 1.31 Å, and 0.37 Å for Ba, Si, and H respectively (VASP POTCAR standard values).

References

(1) Auer, H.; Guehne, R.; Bertmer, M.; Weber, S.; Wenderoth, P.; Hansen, T. C.; Haase, J.; Kohlmann, H. Hydrides of Alkaline Earth–Tetrel (AeTt) Zintl Phases: Covalent Tt–H Bonds from Silicon to Tin. *Inorg. Chem.* **2017**, *56* (3), 1061–1071. https://doi.org/10.1021/acs.inorgchem.6b01944.

(2) Rietveld, H. M. A Profile Refinement Method for Nuclear and Magnetic Structures. *J. Appl. Crystallogr.* **1969**, *2* (2), 65–71. https://doi.org/10.1107/S0021889869006558.

(3) TOPAS (Version 5), General Profile and Structure Analysis Software for Powder Diffraction Data, Bruker AXS, Karlsruhe (Germany), 2014.

(4) Parvanov, V. M.; Schenter, G. K.; Hess, N. J.; Daemen, L. L.; Hartl, M.; Stowe, A. C.; Camaioni, D. M.; Autrey, T. Materials for Hydrogen Storage: Structure and Dynamics of Borane Ammonia Complex. *Dalton Trans.* **2008**, No. 33, 4514–4522. https://doi.org/10.1039/B718138H.

(5) Farla, R.; Bhat, S.; Sonntag, S.; Chanyshev, A.; Ma, S.; Ishii, T.; Liu, Z.; Néri, A.; Nishiyama, N.; Faria, G. A.; Wroblewski, T.; Schulte-Schrepping, H.; Drube, W.; Seeck, O.; Katsura, T. Extreme Conditions Research Using the Large-Volume Press at the P61B Endstation, PETRA III. *J. Synchrotron Rad.* **2022**, *29* (2), 409–423. https://doi.org/10.1107/S1600577522001047.

(6) Dippel, A.-C.; Liermann, H.-P.; Delitz, J. T.; Walter, P.; Schulte-Schrepping, H.; Seeck, O. H.; Franz, H. Beamline P02.1 at PETRA III for High-Resolution and High-Energy Powder Diffraction. *J. Synchrotron Rad.* **2015**, *22* (3), 675–687. https://doi.org/10.1107/S1600577515002222.

(7) Kieffer, J.; Valls, V.; Blanc, N.; Hennig, C. New Tools for Calibrating Diffraction Setups. *J. Synchrotron Rad.* **2020**, *27* (2), 558–566. https://doi.org/10.1107/S1600577520000776.

(8) Shirley, R. *Crysfire 2004: An interactive powder indexing support system. 41 Guildford Park Avenue, Guildford, Surrey, UK, 2004.*

(9) Le Bail, A.; Duroy, H.; Fourquet, J. L. Ab-Initio Structure Determination of $LiSbWO_6$ by X-Ray Powder Diffraction. *Mater. Res. Bull.* **1988**, *23* (3), 447–452. https://doi.org/10.1016/0025-5408(88)90019-0.

(10) Petříček, V.; Dušek, M.; Palatinus, L. Crystallographic Computing System JANA2006: General Features. *Z. Kristallogr. - Cryst. Mater.* **2014**, *229* (5), 345–352. https://doi.org/10.1515/zkri-2014-1737.

(11) Hattori, T.; Sano-Furukawa, A.; Arima, H.; Komatsu, K.; Yamada, A.; Inamura, Y.; Nakatani, T.; Seto, Y.; Nagai, T.; Utsumi, W.; Iitaka, T.; Kagi, H.; Katayama, Y.; Inoue, T.; Otomo, T.; Suzuya, K.; Kamiyama, T.; Arai, M.; Yagi, T. Design and Performance of High-Pressure PLANET Beamline at Pulsed Neutron Source at J-PARC. *Nucl. Instrum. Methods Phys. Res., Sect. A* **2015**, *780*, 55–67. https://doi.org/10.1016/j.nima.2015.01.059.

(12) Ikuta, D.; Ohtani, E.; Sano-Furukawa, A.; Shibazaki, Y.; Terasaki, H.; Yuan, L.; Hattori, T. Interstitial Hydrogen Atoms in Face-Centered Cubic Iron in the Earth's Core. *Sci. Rep.* **2019**, *9*, 7108. https://doi.org/10.1038/s41598-019-43601-z.

(13) Sano-Furukawa, A.; Hattori, T.; Arima, H.; Yamada, A.; Tabata, S.; Kondo, M.; Nakamura, A.; Kagi, H.; Yagi, T. Six-Axis Multi-Anvil Press for High-Pressure, High-Temperature Neutron Diffraction Experiments. *Rev. Sci. Instrum.* **2014**, *85* (11), 113905. https://doi.org/10.1063/1.4901095.

(14) Novak, E.; Haberl, B.; Daemen, L.; Molaison, J.; Egami, T.; Jalarvo, N. Pressure-Induced Phase Transition in Barium Hydride Studied with Neutron Scattering. *Appl. Phys. Lett.* **2020**, *117* (5), 051902. https://doi.org/10.1063/5.0011646.

(15) Brown, J. M. The NaCl Pressure Standard. *J. Appl. Phys.* **1999**, *86* (10), 5801–5808. https://doi.org/10.1063/1.371596.
(16) Matsui, M.; Higo, Y.; Okamoto, Y.; Irifune, T.; Funakoshi, K.-I. Simultaneous Sound Velocity and Density Measurements of NaCl at High Temperatures and Pressures: Application as a Primary Pressure Standard. *Am. Mineral.* **2012**, *97* (10), 1670–1675. https://doi.org/10.2138/am.2012.4136.
(17) Rodríguez-Carvajal, J. FULLPROF: A Program for Rietveld Refinement and Pattern Matching Analysis. In *Abstracts of the satellite meeting on powder diffraction, 15th congress of the IUCr, Toulouse, France*; 1990; p 127.
(18) Hammersley, A. P. *FIT2D*: A Multi-Purpose Data Reduction, Analysis and Visualization Program. *J. Appl. Crystallogr.* **2016**, *49* (2), 646–652. https://doi.org/10.1107/S1600576716000455.
